# Supplementary material for: Arctic sea-ice ridges are biomass hotspots harboring diverse microbial communities
Source: Commun Earth Environ. 2026 Mar 13;7(1):385. doi: 10.1038/s43247-026-03364-8 (PMC13128458; doi:10.1038/s43247-026-03364-8)
Supplement: Supplementary file 2 — Supplementary material [file 43247_2026_3364_MOESM2_ESM.pdf]

Supplementary figures:

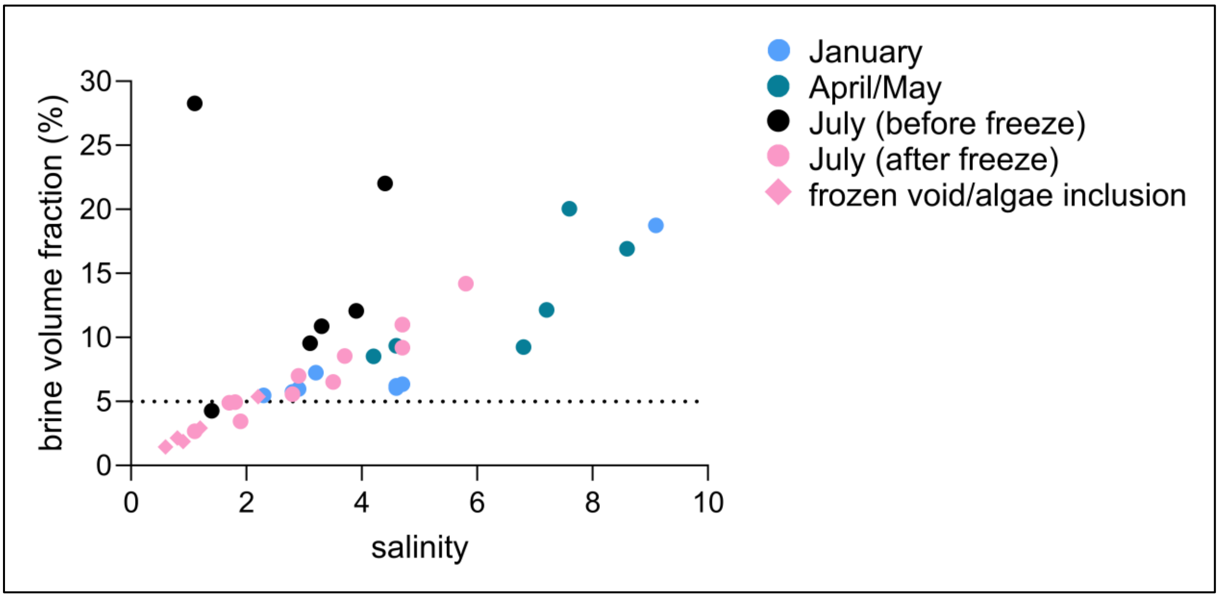

Supplementary Figure 1: Brine volume and bulk salinity of ridge ice samples from January (blue), April, May (turquoise), July before ridge consolidation (black) and July after ridge consolidation (pink). The dashed line indicates the impermeability threshold (<5% brine volume fraction).

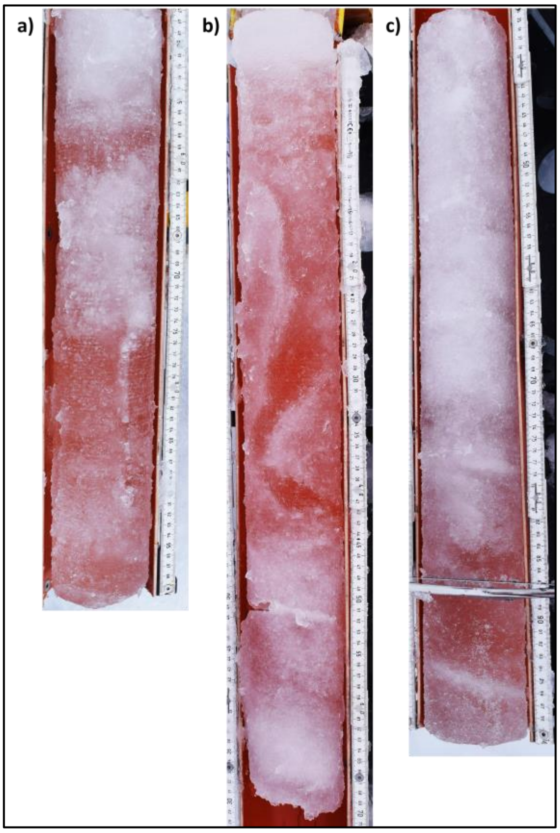

Supplementary Figure 2: Images of parts of the ice cores from R3 coring on a) 11, b) 18 and c) 24 July 2020, showing the contrast between the opaque (former ridge ice rubble) and clear ice, representing the melt water frozen inside water-filled voids. On all three sampling events, the water-filled voids were frozen throughout the entire ridge, except for a core taken at the ridge flank, sampled on 24 July. Scale in cm. Red background from cutting board.

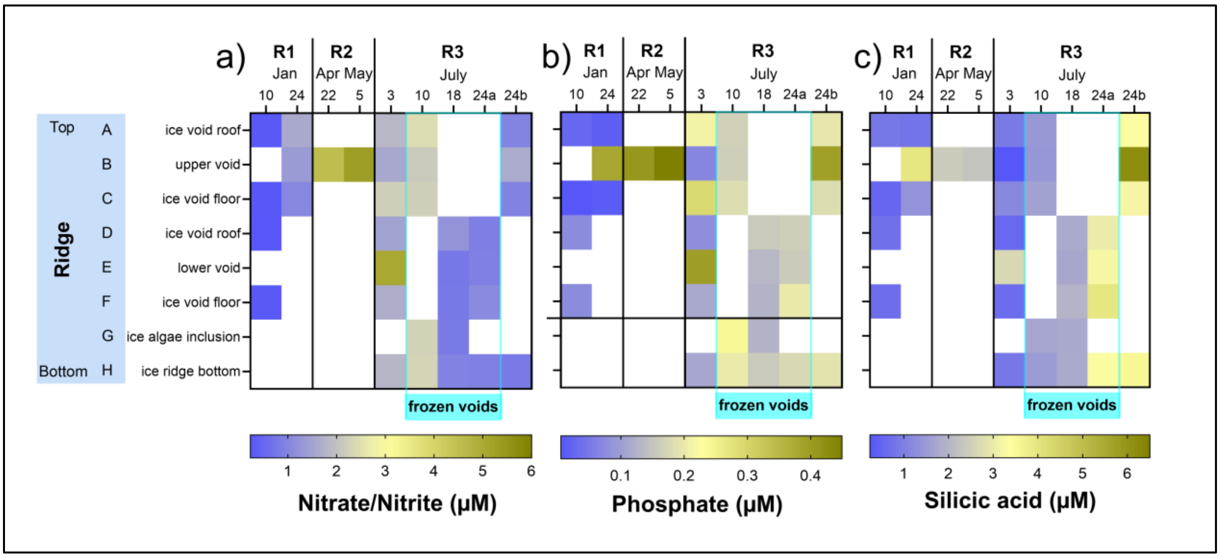

Supplementary Figure 3: Bulk concentrations of a) nitrate/nitrite, b) phosphate, c) silicic acid in samples in the ridge keels (letters A-H indicate the sample location as illustrated in Fig. 2). White background indicates that samples were not taken and the light blue frame (frozen voids) period where water filled voids were frozen.

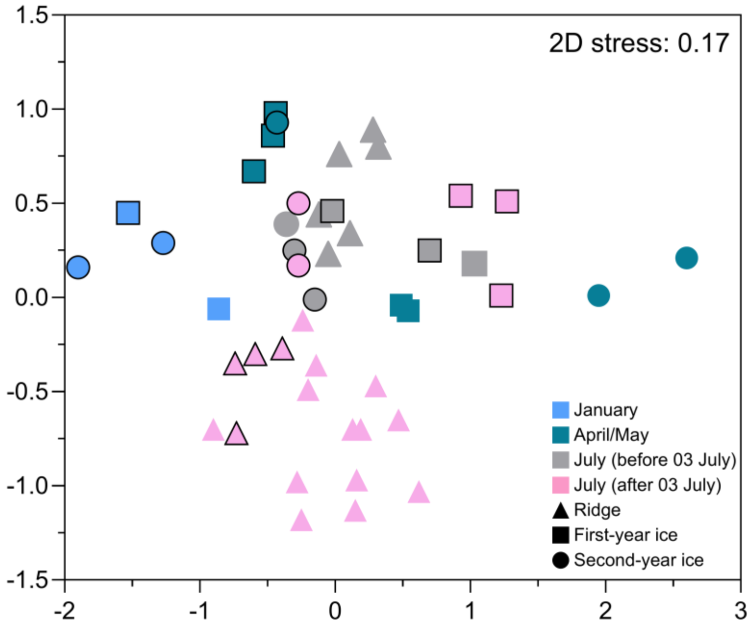

Supplementary Figure 4: Ordination analysis using non-metric Multidimensional Scaling of the eukaryotic (18S rRNA gene) community composition using sequence data from ridge, first-year and second-year ice samples. The sequence data was square-root transformed and sample similarity calculated using Bray-Curtis dissimilarity matrices. Samples are visualized based on season (color) and sample location (shape). Bottom ice samples are indicated with a black frame.

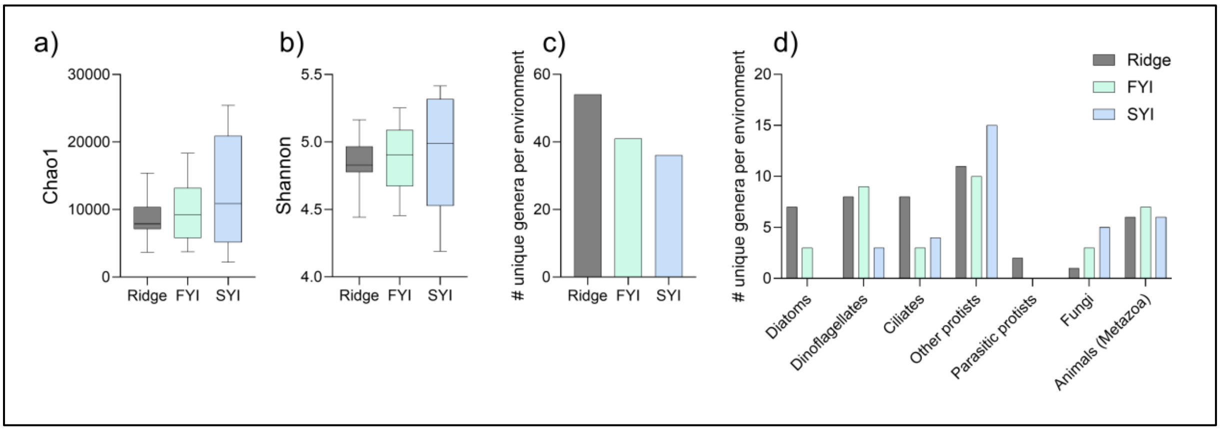

Supplementary Figure 5: Alpha diversity indices, including Chao1 (a) and Shannon diversity (b) presented as median per environment (Ridge, FYI and SYI) from samples taken in April, June and July. Gamma diversity is presented as the number of unique genera per environment group (c) and separated into higher taxonomic groups (d).

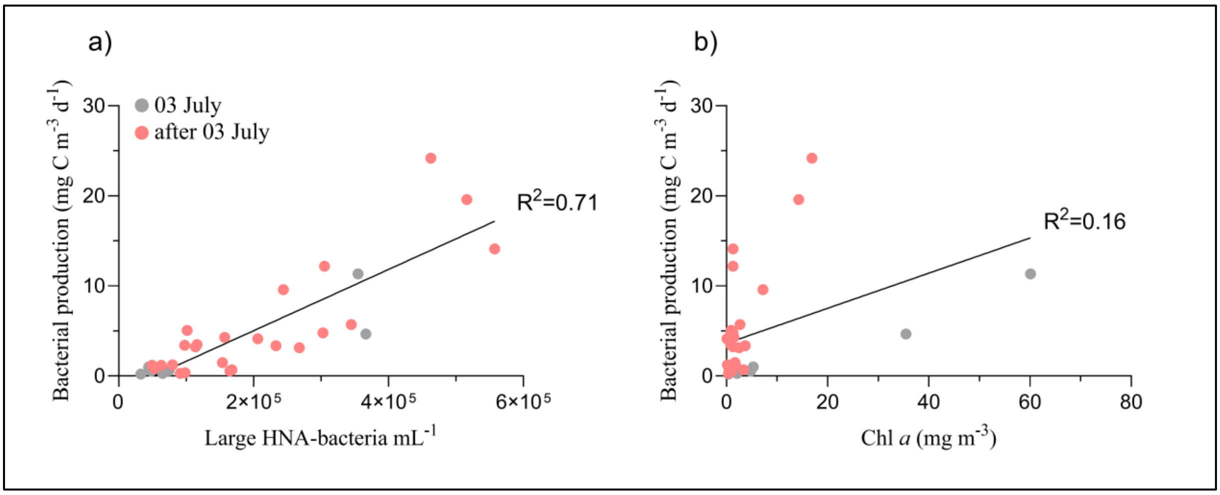

Supplementary Figure 6: Scatter plot showing the relationship between a) large HNA-bacteria and b) chlorophyll a (Chl-a) concentration and bacterial production. Each point represents an individual observation from July when voids were water-filled (grey) or frozen (pink). The black line indicates the best-fit linear regression.

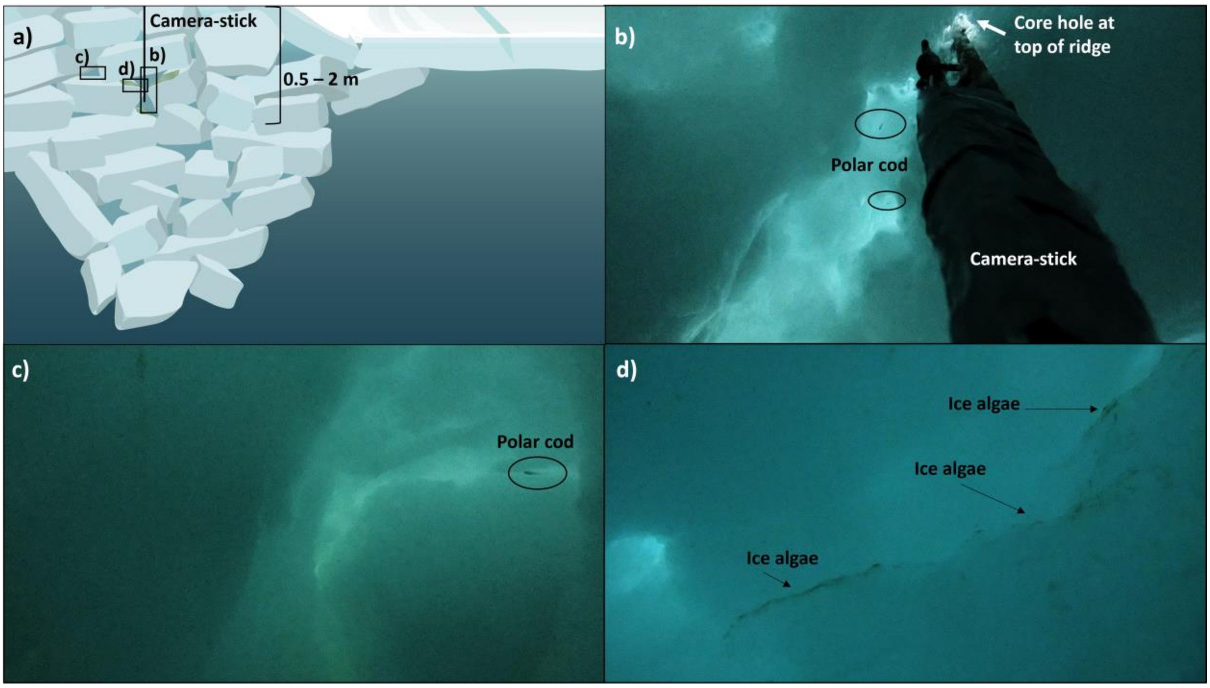

Supplementary Figure 7: Still images from a GoPro video taken during the ridge coring on 3 July, prior to the freezing of the water-filled voids. The schematic (a) highlights where images were taken inside the ridge. The images show the complex structure of the void space and large surface areas with ice-void interfaces (b-d), polar cod in the black circles in b) and c) and algae accumulations on upwards-facing ice surfaces, indicated with black arrows in d).

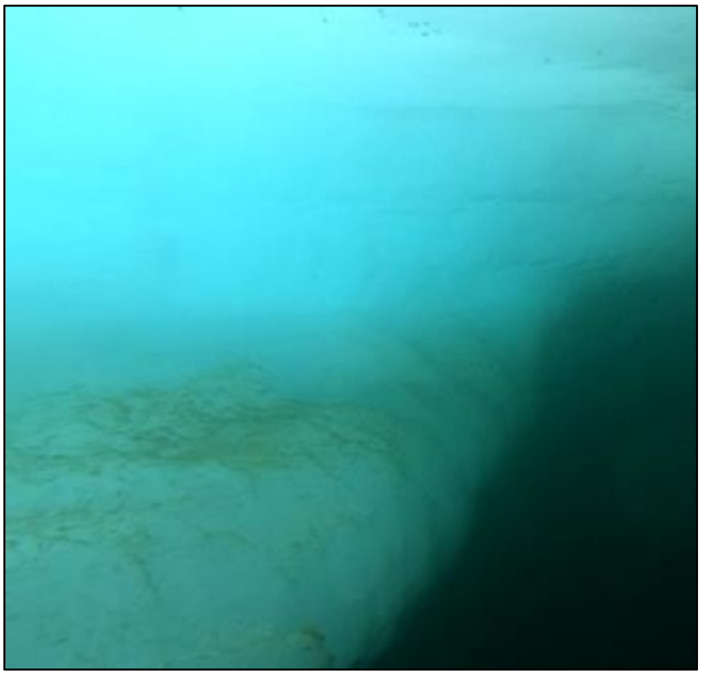

Supplementary Figure 8: Still image of a GoPro video filming under-ice scenery at a rafted level ice site from 19 July. The image shows visibly high concentrations of algae biomass on the upwards-facing ledge ice surface. Camera position is horizontal.

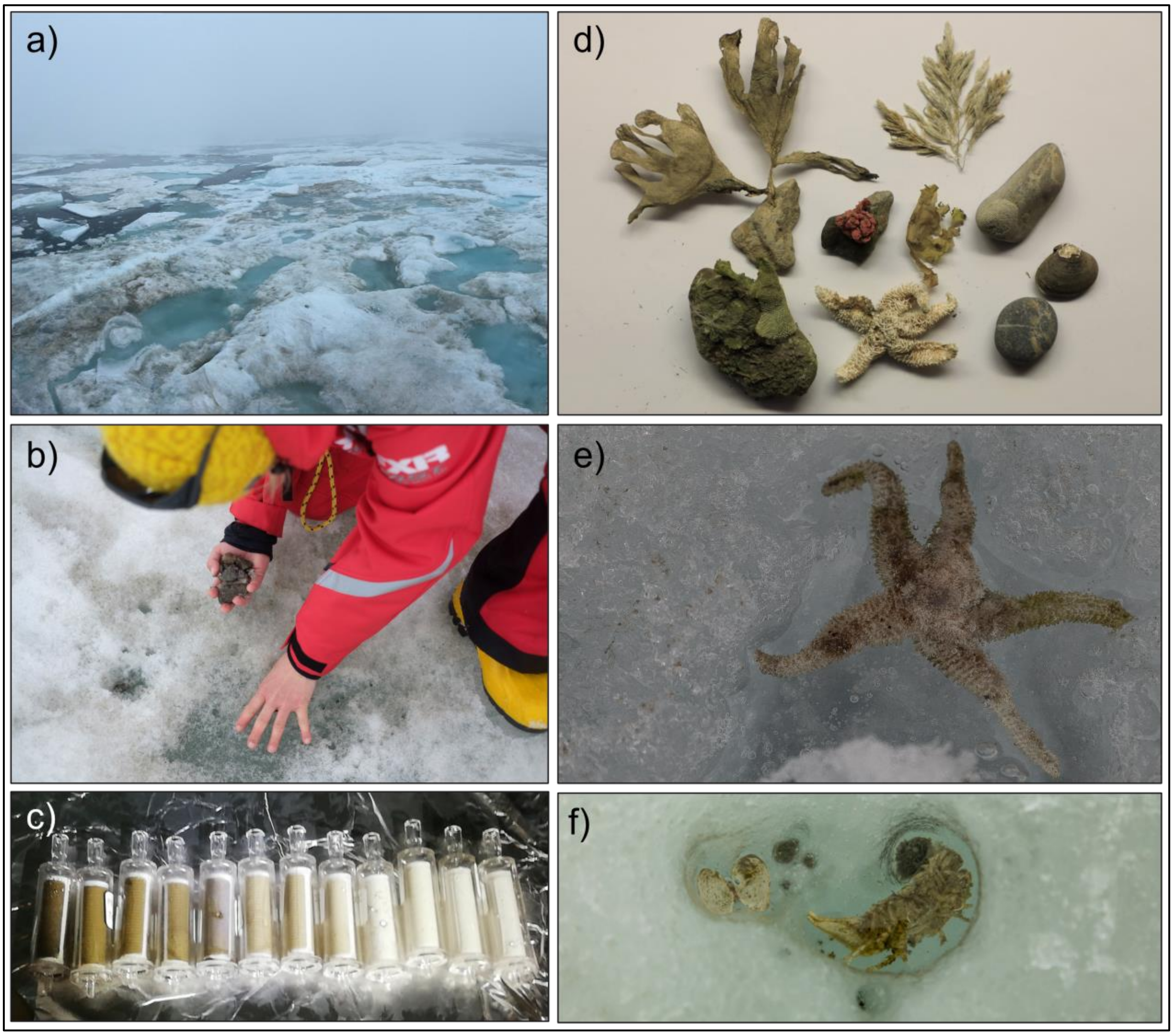

Supplementary Figure 9: A variety of sediment material and large organic structures found in and on the sea ice of the MOSAiC ice floe where the ridges were studied. a) MOASiC ice floe on July 31 2020 showing the dark sedimental material on the melting ice surface (Image: Alfred-Wegener-Institut/ Alessandra D'Angelo (CC-BY 4.0); 31.07.2020); b) close-up of the same material on and in the sea ice, with larger pieces exemplarily shown in the hand (Image: Alfred-Wegener-Institut/Calle Schönning (CC-BY 4.0); 24.07.2020); c) Sterivex filters showing up-concentrated sediment material from filtering melted second year ice samples (Image: Alfred-Wegener-Institut/Oliver Müller (CC-BY 4.0); 08.07.2020); d) selection of benthic flora and fauna found on the MOSAiC ice floe during July (Image: Alfred-Wegener-Institut/Lianna Nixon&Matthew Shupe (CC-BY 4.0); 28.07.2020); e) sea star found on the melting sea ice surface of the MOSAiC ice floe (Image: Alfred-Wegener-Institut/Evgenii Salganik (CC-BY 4.0); 04.07.2020); f) other large organic structures, likely of benthic origin, found in the melting sea ice of the MOSAiC ice floe (Image: Alfred-Wegener-Institut/Lianna Nixon (CC-BY 4.0); 08.07.2020).

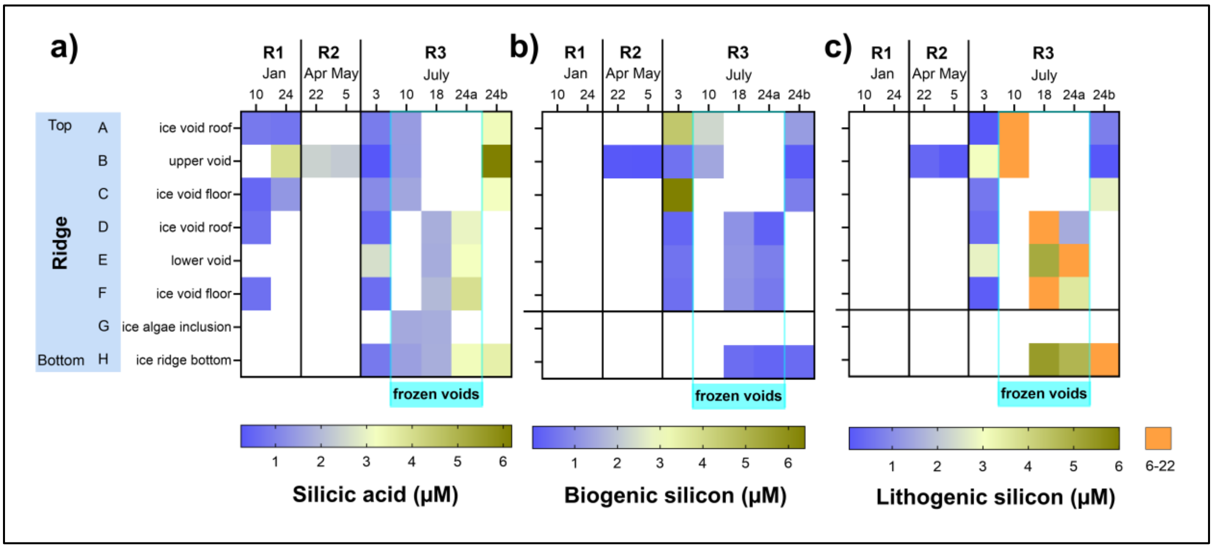

Supplementary Figure 10: Concentrations of a) silicic acid, b) biogenic and c) lithogenic silicon in samples in the ridge keels (letters A-H indicate the sample location as illustrated in Fig. 2). White background indicates that samples were not taken and the light blue frame (frozen voids) is the period where water filled voids were frozen.

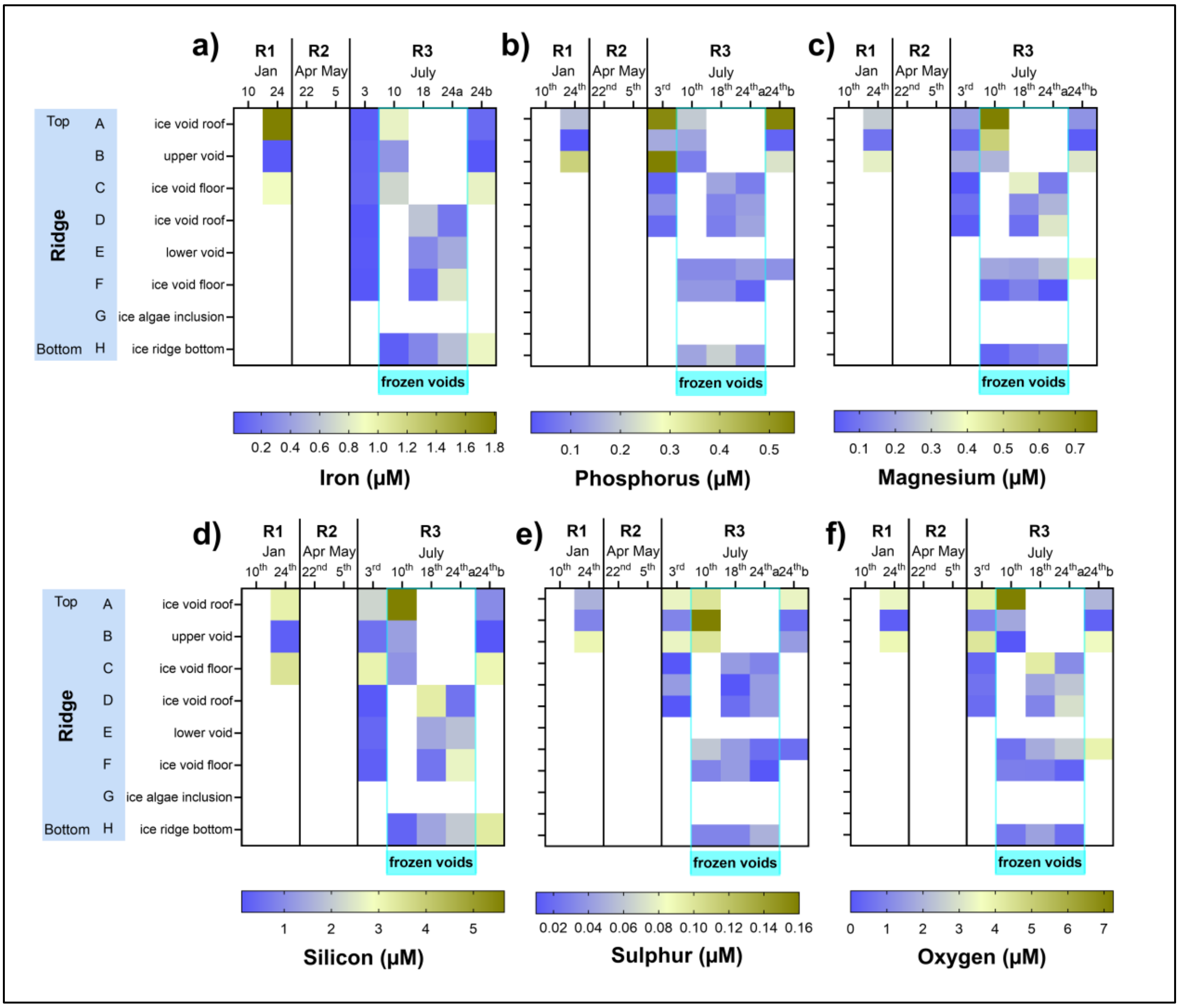

Supplementary Figure 11: Concentrations of particulate a) iron, b) phosphorous, c) magnesium, d) silicon, e) sulphur and f) oxygen measured using X-Ray fluorescence spectroscopy in samples in the ridge keels (letters A-H indicate the sample location as illustrated in Fig. 2). White background indicates that samples were not taken and the light blue frame (frozen voids) is the period where water filled voids were frozen.

Supplementary tables:

Supplementary table 1: Maximum chlorophyll *a* (Chl-a) concentration, bacteria and phytoplankton abundance in different ice types and seawater.

Sample (number of samples)	Chl-a ($mg\ m^{-3}$)	Bacteria (cells x 10^6 mL^{-1})	Picophytoplankton (cells x 10^4 mL^{-1})	Nanophytoplankton (cells x 10^4 mL^{-1})
Ridge (n=57)	**60.1**	0.68	0.57	0.60
FYI* (n=115)	7.9	**1.05**	0.43	**0.71**
SYI* (n=159)	5.3	0.86	0.11	0.37
Seawater (n=35)	1.0	0.62	**1.27**	0.14

The sampling dates are given in Figure 4. Seawater samples are from <200 m depth.
*For first-year (FYI) and second-year ice (SYI) the mean value of the bottom 10 cm was calculated since all ridge ice samples were taken in 10 cm sections. Bold values indicate the highest values within the different environments.

Supplementary table 2: Pairwise PERMANOVA results based on Bray-Curtis dissimilarity matrices comparing microbial community compositions across clusters using 16S rRNA gene data (prokaryotes) and 18S rRNA gene data (eukaryotes).

**Clusters - 16S rRNA genes - Ridge**			**SumsOfSqs**	**F.Model**	**R2**	**p**	**p adjusted**
Winter	vs	Water-filled_void	1.66	18.41	0.72	0.007	**0.042**
Winter	vs	UiW	1.28	15.17	0.60	0.006	**0.036**
Winter	vs	Frozen_void	2.17	11.84	0.38	0.001	**0.006**
Water-filled_void	vs	UiW	1.57	13.08	0.54	0.001	**0.006**
Water-filled_void	vs	Frozen_void	0.95	4.81	0.19	0.001	**0.006**
UiW	vs	Frozen_void	2.52	13.87	0.38	0.001	**0.006**
**Clusters - 18S rRNA genes - Ridge**							
Winter	vs	Water-filled_void	1.21	7.14	0.50	0.01	0.06
Winter	vs	UiW	1.32	10.11	0.50	0.006	**0.036**
Winter	vs	Frozen_void	1.55	6.37	0.25	0.002	**0.012**
Water-filled_void	vs	UiW	1.56	11.81	0.52	0.001	**0.006**
Water-filled_void	vs	Frozen_void	1.03	4.33	0.18	0.001	**0.006**
UiW	vs	Frozen_void	2.72	12.77	0.36	0.001	**0.006**
**Clusters - 18S rRNA genes - Ridge, FYI & SYI**							
Water-filled_void	vs	Level_bottom	0.53	3.71	0.24	0.003	**0.045**
Water-filled_void	vs	Level_interior	0.75	4.07	0.25	0.002	**0.03**
UiW	vs	Level_bottom	2.38	20.19	0.57	0.001	**0.015**
UiW	vs	Level_interior	2.23	14.68	0.49	0.001	**0.015**
Frozen_void	vs	Level_bottom	1.18	6.91	0.22	0.001	**0.015**
Frozen_void	vs	Level_interior	1.09	5.68	0.19	0.001	**0.015**
Level_bottom	vs	Level_interior	1.13	6.34	0.28	0.001	**0.015**

Each row shows the comparison between two clusters. Reported statistics include: Sums of Squares (SumsOfSqs), F-statistic (F.Model), coefficient of determination (R^2), unadjusted p-values (p), and adjusted p-values using the Bonferroni correction for multiple comparisons (p adjusted). Statistically significant differences (p adjusted < 0.05) are indicated in bold.

Supplementary table 3: METABOLIC analysis of MAGs from bottom and interior ridge ice samples

KEGG Module-ID	KEGG Module	Ridge-bottom-ice (n=222) %	Ridge-internal-ice (n=539) %
M00122	Cobalamin biosynthesis, cobyrinate a,c-diamide => cobalamin	12	19
M00899	Thiamine salvage pathway, HMP/HET => TMP	1	7

Percentage of MAGs with hits for genes as part of KEGG modules encoding enzymes involved in algae-growth promoting cofactor pathways. n = number of MAGs

Supplementary table 4: METABOLIC analysis of MAGs from interior ridge ice samples comparing differences between water-filled and frozen voids

Category	Function	Gene abbreviation	Water-filled void (n=273)	Frozen void (n=266)
Amino acid utilization	Histidinol-phosphate/aromatic aminotransferase	histidinol-phosphate/aromatic aminotransferase	60	76
	Serine-pyruvate aminotransferase/archaeal aspartate aminotransferase	serine-pyruvate aminotransferase/archaeal aspartate aminotransferase	18	25
	4-aminobutyrate aminotransferase and related aminotransferases	4-aminobutyrate aminotransferase and related aminotransferases	7	10
Aromatics degradation	Phenol => Benzoyl-CoA	ubiX||bsdC	23	29
	Protocatechuate/Catechol degradation	catA	0	1
Complex carbon degradation	Amylolytic enzymes	pullulanase	6	15
	Amylolytic enzymes	glucoamylase	1	3
Fermentation	Alcohol utilization	adh	0	3
	Lactate utilization	ldh	1	3
	Pyruvate <=> acetyl-CoA + formate	pflD	1	3
Nitrogen cycling	Nitric oxide reduction	norBC	3	11
	Nitrate reduction	napAB||narGH	2	9
	Nitrous oxide reduction	nosDZ	1	6
	Nitrite reduction	nirKS||octR	3	5

Percentage of MAGs with hits for genes encoding proteins sorted according to different metabolic categories. The ridge ice samples were collected when voids were water-filled and frozen. n = number of MAGs

Supplementary table 5: dbCAN analysis of MAGs from interior ridge ice samples comparing differences between water-filled and frozen voids

Substrate	Water-filled void (n=270) %	Frozen void (n=266) %
sucrose	55.6	69.9
trehalose	11.9	16.5
sialic acid	1.5	4.1
glucosylglycerate	0	2.6

chitooligosaccharide	9.3	10.9
rhamnose	6.7	7.1
fucose	2.6	3
alpha-rhamnoside	2.2	2.6
raffinose	3	0.8
exo-polysaccharide	26.7	40.2
fructan	7	19.5
alpha-glucan	37.4	46.6
starch	42.6	51.5
glycogen	42.2	51.1
arabinan	2.6	4.5
beta-glucuronan	1.9	3
beta-galactan	23.7	24.1
glycolipid	0.7	0
beta-glucan	67	63.2
host glycan	49.6	44.4
beta-mannan	21.5	15
pectin	4.1	13.9
xyloglucan	30.7	39.1
cellulose	21.9	26.3
alginate	0.7	4.9
chitin	91.1	94
xylan	50.4	50
chitosan	0.4	0
alpha-mannan	1.9	1.1
polyphenol	11.5	21.8
lignin	16.7	19.5
fucoidan	0	1.1
agarose	0	0.4
carrageenan	0	0.8

Percentage of MAGs with hits for genes encoding proteins sorted according to different substrates. The ridge ice samples were collected when voids were water-filled and frozen. n = number of MAGs

Supplementary table 6: METABOLIC analysis of MAGs from ridge bottom and interior ridge ice samples when voids were water-filled and frozen.

Category	Ridge-bottom-ice (n=24)	Water-filled void (n=44)	Frozen void (n=71)
Amino acid utilization	11	17	31
Ethanol fermentation	40	37	48
Fatty acid degradation	13	15	28
Aromatics degradation	15	24	51
Complex carbon degradation	12	18	30
Fermentation	10	18	28
Carbon degradation (sum of all above)	12	18	31

Percentage of MAGs with hits for genes encoding proteins linked to different carbon degradation categories that were classified as Gammaproteobacteria. The ridge ice samples were collected when voids were water-filled and frozen. n = number of MAGs assigned to Gammaproteobacteria

Supplementary table 7: METABOLIC analysis showing carbohydrate-active enzymes linked to complex carbon degradation of 12 MAGs from ridge samples assigned to the genus *Colwellia.*

			MAG #											
**Category**	**Function**	**Gene abbreviation**	**1**	**2**	**3**	**4**	**5**	**6**	**7**	**8**	**9**	**10**	**11**	**12**
Complex carbon degradation	Cellulose degrading	cellobiosidase	-	-	-	-	-	-	-	-	-	-	-	-
		cellulase	-	-	-	-	-	+	-	+	-	+	-	-
		beta-glucosidase	+	+	+	+	-	+	-	+	+	+	-	+
	Hemicullulose debranching	arabinosidase	-	-	-	-	-	-	-	-	-	-	-	-
		beta-glucuronidase	-	-	-	-	-	-	-	-	-	-	-	-
		alpha-L-rhamnosidase	-	-	-	-	-	-	-	-	-	-	-	-
	Endohemi-cellulases	mannan endo-1,4-beta-mannosidase	-	-	-	-	-	-	-	-	-	-	-	-
		alpha-D-xyloside xylohydrolase	-	-	-	-	-	-	-	-	-	-	-	-
	Other oligosaccharide degrading	beta-xylosidase	-	-	-	-	-	-	-	-	-	-	-	-
		beta-mannosidase	-	-	-	-	-	-	-	-	-	+	-	-
		beta-galactosidase	+	-	-	-	-	+	-	-	-	-	+	-
	Amylolytic enzymes	alpha-amylase	-	-	-	+	-	+	-	-	-	-	-	-
		glucoamylase	-	-	-	-	-	-	-	-	-	-	-	-
		pullulanase	+	-	+	-	-	+	-	-	-	+	-	+
		isoamylase	+	-	-	+	-	-	-	+	+	-	-	-
	Chitin degrading	chitinase	-	-	-	-	-	+	-	-	+	+	-	-
		hexosaminidase	+	+	+	+	+	+	-	+	+	+	-	+

Presence (+) / absence (-) matrix for genes encoding 17 carbohydrate-active enzymes linked to complex carbon degradation.

Supplementary methods:

Chlorophyll *a* correction factor

Chlorophyll *a* samples collected from the ridge (R3) in July 2020 were not treated according to standard protocols. At the end of the campaign, after being kept frozen at -80°C, the filters were, due to a mistake, dried overnight at 60 °C and upon realization of the mistake the following day, placed back into the -80°C freezer and kept frozen until analysis. To test how this procedure impacted Chl-a concentrations, laboratory cultures of mixed pennate diatoms (Supplementary Fig. 12a) and of the centric diatom *Attheya septentrionalis* (Supplementary Fig. 12b) from the Arctic (northern Barents Sea) were used. Six replicates of different volumes (0.1, 1, 2.5, 5, 10 and 15 mL) of the cultures were filtered

on GF/F 25 mm filters (Whatman) using low-pressure vacuum. For each volume one set of three replicates was treated according to standard protocol and kept frozen at -80°C, while the other set of three replicates was first frozen at -80°C for three weeks, then dried overnight at 60 °C and placed back into the -80°C freezer the following day to replicate the procedure from the field. For the measurement, all Chl-a filters were extracted in 90% acetone overnight at 4°C and subsequently analyzed on a Turner Design 10-AU fluorometer (Turner Designs, USA), including an acidification step (3 drops of 1 M HCl) to determine phaeopigments [1]. The measurements showed a consistently lower value in the dried treatment than the standard treatment, independent of the Chl-a concentration. A linear regression analysis showed a slope of 0.55 and 0.58 for the diatom mix and *Attheya*, respectively. We used the average of both slopes (0.56) to retrieve a correction factor (1.8) that we applied to the field samples that were dried overnight at 60 °C. Similarly, the analysis of phaeopigment concentrations showed a consistently lower value in the dried treatment than the standard treatment, independent of the concentration (Supplementary Fig. 12c and d). The linear regression analysis showed that the drying impacted phaeopigment concentrations to a lesser degree in the laboratory culture of the centric diatom *Attheya septentrionalis* than in the culture of mixed pennate diatom.

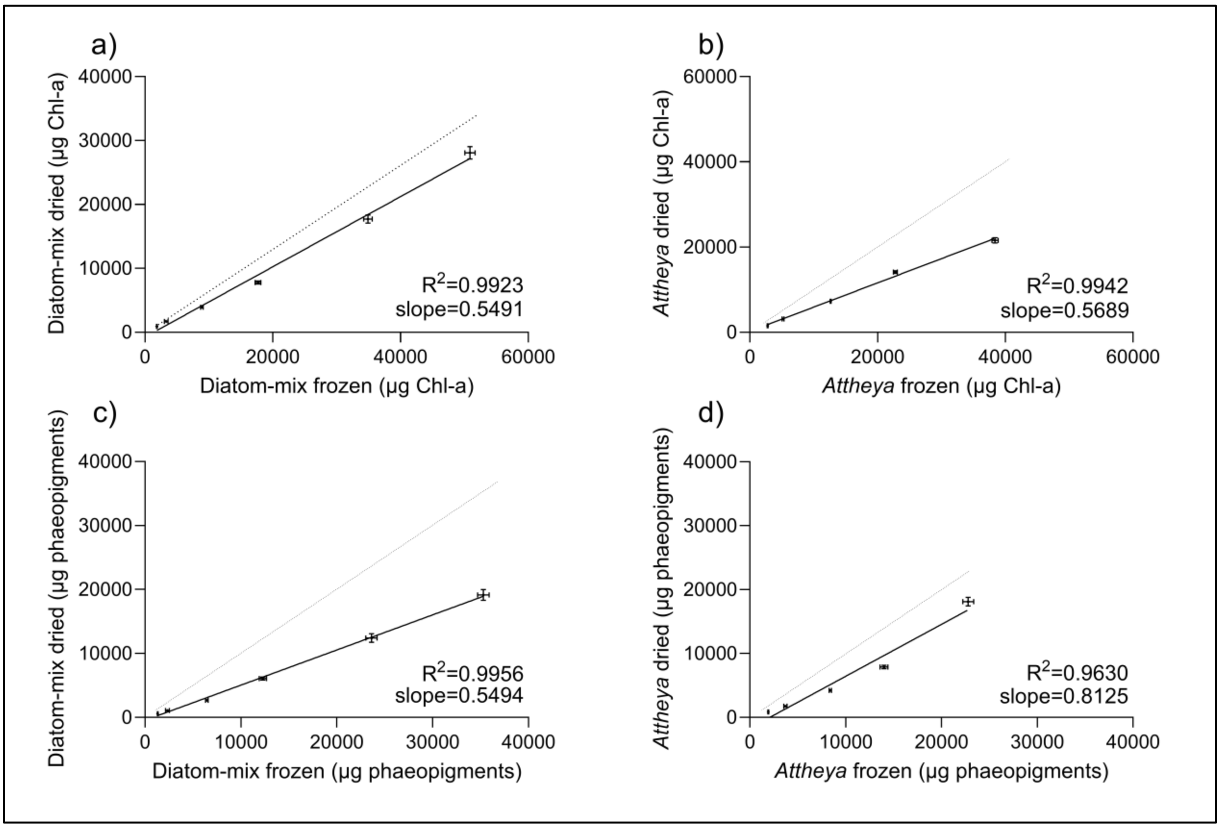

Supplementary Figure 12: The impact of overnight drying (60 °C) on chlorophyll *a* concentrations using laboratory cultures of mixed pennate diatoms (a and c) and of the centric diatom *Attheya septentrionalis* (b and d) from the Arctic (northern Barents Sea). Six replicates of different volumes (0.1, 1, 2.5, 5, 10 and 15 mL) of the cultures were filtered on GF/F 25 mm filters (Whatman) using low-pressure vacuum. For each volume one set of three replicates was treated according to standard protocol and kept frozen at -80°C, while the other set of three replicates was first frozen at -80°C for three weeks, then dried overnight at 60 °C and placed back into the -80°C freezer the following day to replicate the procedure from the field.

Elemental composition of particles (XRF)

For the analysis of total particulate element concentrations, 0.25–1 L of melted sea ice or water samples were filtered on Whatman® Nucleopore™ polycarbonate (PC) filters (47 mm in diameter, 0.6 µm pore size) using ≤30 KPa vacuum pressure. After all the sample water went through, the filter was rinsed with 5 ml of MQ water to remove any salts, air-dried and kept dry at room temperature in petri slides until analysis. Total particulate concentrations of P, S, O, Si, Fe and Mg were measured by wavelength dispersive X-Ray fluorescence spectroscopy (WDXRF) using a Bruker® AXE S4 pioneer XRF instrument. The concentrations for the elements were calculated based on the calibration parameters and detection limits explained in Paulino et al., 2013[2]. The instrument provides a bulk amount of particulate element concentration and does not differentiate between different chemical forms. Three

blank filters from each new batch of PC filters and three filters with MQ-water were used as references prior to the sample measurements for correction.

Flow cytometry gating strategy

Photosynthetic organisms (Supplementary Fig. 13a and b) were discriminated according to their grouping on either BL3 (red fluorescence) vs. BL2 (orange fluorescence) or BL3 (red fluorescence) vs. SSC (side scatter), similar to gating strategies as presented in Thyssen et al., 2022[3]. The black frame highlights all photosynthetic organisms in the size range of 1 and 20 µm. Different size groups are indicated by the coloured frames (red: picophytoplankton [1-2 µm]; purple: small nanophytoplankton [2-5 µm] and green: large nanophytoplankton [5-20 µm]). Bacteria (Supplementary Fig. 13c) were discriminated using green fluorescence (BL1) vs. side scatter (SSC) as described in Marie et al. (1999)[4] and grouped into low nucleic acid (LNA) containing bacteria (green) and high nucleic acid (HNA) containing bacteria (blue). Different size groups of virus particles are highlighted in pink, grey and yellow for reference, but are not included in this study.

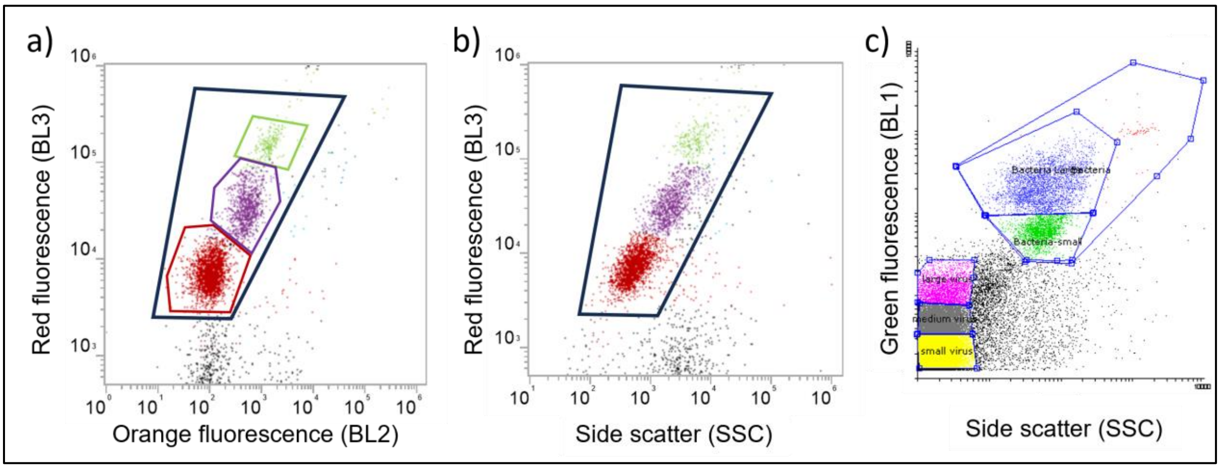

Supplementary Figure 13: Biparametric flow cytometry plots with the applied gating strategy for the different phytoplankton groups (a-b) and bacteria (c).

Supplementary references:

1. Knap, A., Michaels, A., Close, A., Ducklow, H. & Dickson, A. Protocols for the Joint Global Ocean Flux Study (JGOFS) Core Measurements. (1996).

2. Paulino, A. I., Heldal, M., Norland, S. & Egge, J. K. Elemental stoichiometry of marine particulate matter measured by wavelength dispersive X-ray fluorescence (WDXRF) spectroscopy. *J. Mar. Biol. Assoc. United Kingdom* **93**, 2003–2014 (2013).

3. Thyssen, M. *et al.* Interoperable vocabulary for marine microbial flow cytometry. *Front. Mar. Sci.* **9**, 975877 (2022).

4. Marie, D., Brussaard, C. P. D., Thyrhaug, R., Bratbak, G. & Vaulot, D. Enumeration of Marine Viruses in Culture and Natural Samples by Flow Cytometry. *Appl. Environ. Microbiol.* **65**, 45–52 (1999).
