# Supplementary material for: Arctic sea-ice ridges are biomass hotspots harboring diverse microbial communities
Source: Commun Earth Environ. 2026 Mar 13;7(1):385. doi: 10.1038/s43247-026-03364-8 (PMC13128458; doi:10.1038/s43247-026-03364-8)
Supplement: Supplementary file 3 — nr-reporting-summary [file 43247_2026_3364_MOESM3_ESM.pdf]

nature portfolio

Corresponding author(s): *Double-anonymous peer review submissions: write DAPR and your manuscript number here instead of author names.*

Last updated by author(s): Nov 11, 2025

Reporting Summary

Nature Portfolio wishes to improve the reproducibility of the work that we publish. This form provides structure for consistency and transparency in reporting. For further information on Nature Portfolio policies, see our Editorial Policies and the Editorial Policy Checklist.

Statistics

For all statistical analyses, confirm that the following items are present in the figure legend, table legend, main text, or Methods section.

n/a	Confirmed	
☒	☐	The exact sample size (*n*) for each experimental group/condition, given as a discrete number and unit of measurement
☐	☒	A statement on whether measurements were taken from distinct samples or whether the same sample was measured repeatedly
☐	☒	The statistical test(s) used AND whether they are one- or two-sided<br>*Only common tests should be described solely by name; describe more complex techniques in the Methods section.*
☐	☒	A description of all covariates tested
☐	☒	A description of any assumptions or corrections, such as tests of normality and adjustment for multiple comparisons
☐	☒	A full description of the statistical parameters including central tendency (e.g. means) or other basic estimates (e.g. regression coefficient) AND variation (e.g. standard deviation) or associated estimates of uncertainty (e.g. confidence intervals)
☐	☒	For null hypothesis testing, the test statistic (e.g. *F*, *t*, *r*) with confidence intervals, effect sizes, degrees of freedom and *P* value noted<br>*Give P values as exact values whenever suitable.*
☒	☐	For Bayesian analysis, information on the choice of priors and Markov chain Monte Carlo settings
☒	☐	For hierarchical and complex designs, identification of the appropriate level for tests and full reporting of outcomes
☐	☒	Estimates of effect sizes (e.g. Cohen's *d*, Pearson's *r*), indicating how they were calculated

Our web collection on statistics for biologists contains articles on many of the points above.

Software and code

Policy information about availability of computer code

Data collection	n/a
Data analysis	Statistical analyses were done using the R (v.4.4.5) package vegan (v.2.6-8) and the programs primer-e version 6 (Plymouth, UK) and Canoco 5 (ter Braak and Šmilauer, 2012). Among others, Bray-Curtis dissimilarities, principal component analysis (PCA) and linear regressions were calculated using these tools. The connections between environmental variables were analyzed using PCA, performed on normalized data using centred log-ratio transformation (Gloor and Reid, 2016). The coordinates of the scores and loadings for the PCA plots were illustrated using GraphPad Prism version 10.1.2 for Windows (GraphPad Software, USA, www.graphpad.com). For the ordination analysis (NMDS) of the sequencing data, the relative abundance at genus level was used and the table was normalized using square-root transformation, before Bray-Curtis dissimilarity was calculated to assess community similarity among samples.<br>The amplicon sequence data was processed using DADA2 (Divisive Amplicon Denoising Algorithm 2, version 1.32.0) in R (version 4.4.1). In short, sequences were trimmed, filtered based on quality scores, dereplicated and amplicon sequencing variants (ASVs) inferred. Forward and reverse reads were denoised and merged before chimeric sequences were removed, and taxonomy was assigned using the 16S silva database (version 138) and the 18S pr2 database (version 4.12.0) for 16S and 18S sequences, respectively. The resulting ASV tables (16S and 18S), along with taxonomic annotations, were used for all subsequent diversity and community composition analyses. Relative abundance was calculated per sample by dividing each ASV count by the total number of reads.<br>Metagenome data was assembled using the JGI MAP pipeline, where samples were filtered for quality with BBDuk, error corrected using BBCMS, assembled using SPAdes, and reads mapped back to contigs using BBMap (v38.86). Metagenomic data were analyzed to compare functional gene profiles across samples. Annotated gene abundances were grouped according to Clusters of Orthologous Groups (COG) pathways. Gene abundance tables were normalized to account for differences in sequencing depth. Principal Component Analysis (PCA) was used to explore patterns of variation in COG pathway abundance among samples. Metagenome assembled genomes (MAGs) recovered from

the HAVOC metagenomes (PRJNA1160706), as described in Boulton et al., 2025, were analyzed using METABOLIC v4.0 to infer metabolic potential and using dbCAN4 to identify and annotate carbohydrate-active enzymes using the Carbohydrate-Active EnZymes database (CAZy).

For manuscripts utilizing custom algorithms or software that are central to the research but not yet described in published literature, software must be made available to editors and reviewers. We strongly encourage code deposition in a community repository (e.g. GitHub). See the Nature Portfolio guidelines for submitting code & software for further information.

Data

Policy information about availability of data

All manuscripts must include a data availability statement. This statement should provide the following information, where applicable:

- Accession codes, unique identifiers, or web links for publicly available datasets
- A description of any restrictions on data availability
- For clinical datasets or third party data, please ensure that the statement adheres to our policy

All environmental data, including nutrients, POC, biogenic and lithogenic silica, elemental composition of particles, chlorophyll a, bacterial production, flow cytometry and light microscopy counts (simplified) from ridge samples can be found compiled in one data set on PANGAEA (Müller et al., 2025; https://doi.org/10.1594/PANGAEA.983955).
Non-ridge data can be found as follows:
Dissolved nutrients in the water column can be found in Torres-Valdés et al. (2024; https://doi.org/10.1594/PANGAEA.966217). Biogenic and lithogenic silica in the level ice and water column can be found in Lemke et al. (2024; https://doi.org/10.1594/PANGAEA.971253). Chlorophyll a concentrations in level ice and water column can be found in van Leeuwe et al. (2024; https://doi.org/10.1594/PANGAEA.967448) and Hoppe et al. (2023; https://doi.org/10.1594/PANGAEA.963277), flow cytometry in water column and level ice is available in Müller et al. (2023; https://doi.org/10.1594/PANGAEA.963430) and Müller et al. (2023; https://doi.org/10.1594/PANGAEA.963560).

The sequencing raw data is available at the European Nucleotide Archive (ENA); from ridge samples under accession number PRJEB90852 and for non-ridge samples under PRJNA895866. Metagenome data is available under JGI Project 1454842 and Metagenome assembled genomes (MAGs) recovered from the HAVOC metagenomes under accession number PRJNA1160706.

Light microscopy counts (taxonomy) of sea ice (including ridges) can be found in Assmy et al. (2023; https://doi.org/10.1594/PANGAEA.957637) and for pelagic samples (incl. ridge voids) in Assmy et al. (2023; https://doi.org/10.1594/PANGAEA.957640). Biogenic silica data are available in Lemke et al. (2024; https://doi.org/10.1594/PANGAEA.971253).
For R1, drilling data can be found in Salganik et al. (2023; https://doi.org/10.1594/PANGAEA.960347); coring data in Salganik et al. (2023; https://doi.org/10.1594/PANGAEA.962542); ice mass balance buoy data in Granskog et al. (2020; https://doi.org/10.1594/PANGAEA.924269), Granskog et al. (2021; https://doi.org/10.1594/PANGAEA.938354) and in Salganik et al. (2023; https://doi.org/10.1594/PANGAEA.964023).
For R2, the coring data can be found in Salganik et al. (2025, https://doi.pangaea.de/10.1594/PANGAEA.979884).
For R3, drilling data can be found in Salganik et al. (2023b; https://doi.org/10.1594/PANGAEA.953880), and the ice mass balance buoy data in Granskog et al. (2021; https://doi.org/10.1594/PANGAEA.938354);
Level first-year ice coring data can be found in Oggier et al. (2024; https://doi.org/10.1594/PANGAEA.971385) and level second-year ice in Oggier et al. (2025; https://doi.org/10.1594/PANGAEA.974764). Multibeam sonar data can be found in Anhaus et al. (2024; https://doi.org/10.1594/PANGAEA.971872), the airborne laser scanner measurements can be found in Hutter et al. (2023; https://doi.org/10.1594/PANGAEA.950896), the helicopter-borne RGB orthomosaics can be found in Neckel et al. (2023; (https://doi.org/10.1594/PANGAEA.949433), and the core hydrographic data can be found in Schulz et al. (2023b; https://doi.org/10.18739/A21J9790B).

Research involving human participants, their data, or biological material

Policy information about studies with human participants or human data. See also policy information about sex, gender (identity/presentation), and sexual orientation and race, ethnicity and racism.

Reporting on sex and gender	This research does not involve human participants, their data, or biological material
Reporting on race, ethnicity, or other socially relevant groupings	This research does not involve human participants, their data, or biological material
Population characteristics	This research does not involve human participants, their data, or biological material
Recruitment	This research does not involve human participants, their data, or biological material
Ethics oversight	This research does not involve human participants, their data, or biological material

Note that full information on the approval of the study protocol must also be provided in the manuscript.

Field-specific reporting

Please select the one below that is the best fit for your research. If you are not sure, read the appropriate sections before making your selection.

☐ Life sciences ☐ Behavioural & social sciences ☒ Ecological, evolutionary & environmental sciences

For a reference copy of the document with all sections, see nature.com/documents/nr-reporting-summary-flat.pdf

Ecological, evolutionary & environmental sciences study design

All studies must disclose on these points even when the disclosure is negative.

Study description	The study consists of different field data collected during the MOSAiC campaign with specific focus on sea ice ridges. The aim of the study was to combine biological, biogeochemical and physical data of pressure ridges to provide a detailed ecological inventory of different ridge habitats and how they change from winter to summer and a particular focus on microbial processes.
Research sample	All samples were collected directly on the sea ice, both sea ice ridges and level first-year and second-year ice for comparison. Ice cores were extracted with a 9-cm (Mark II) internal diameter ice corer (Kovacs Enterprise, USA) and were cut into 10 cm long sections for biogeochemical variables in the field and collected in sterile plastic bags, with the focus on the three habitats: the ice of the roof and the floor of water-filled voids, the bottom of the ridge, and, when present, the frozen void and algae inclusions. When possible, the water (20–30 L) inside the voids, below the ridge and below level ice, was sampled using a manual bilge pump with a silicon tube with a diameter of 20 mm into prewashed polyethylene containers. Seawater samples for comparison were collected from RV Polarsterns CTD rosette system. From both melted sea ice and water samples, sub-samples were taken for determination of inorganic nutrients, biogenic silica (BSi), particulate organic carbon (POC), elemental composition of particles (XRF), chlorophyll a (Chl-a), bacterial production (BP) and abundance and diversity estimates of protists and bacteria through flow cytometry (FCM), light microscopy and molecular analysis.
Sampling strategy	The yearlong MOSAiC ice drift (October 2019 to September 2020; e.g. Nicolaus et al., 2022; Fong et al., 2024), with the research vessel Polarstern served as the base for detailed and interdisciplinary observations of ridges. During the drift, three different ridges were sampled at different times of the year. The changes between ridges were necessary, as logistical challenges and ice dynamics prevented the sampling of the same ridge throughout the entire period (see below), highlighting the difficulties associated with studying ridges. The first ridge (R1) was investigated in winter, the second ridge (R2) was investigated in spring, and the third ridge (R3) was investigated in summer. Based on their macrostructural physical properties, the three ridges were similar in characteristics. They formed during the MOSAiC drift (similar age) and were composed of thin ice blocks, with similar sail heights (1–2 m) and average keel depths (3.2–4.3 m). Comparative data from first-year, second-year level ice (FYI and SYI) and seawater used in this study is from ice cores and seawater taken as part of the MOSAiC main programme, for further details see Nicolaus et al. (2022) and Fong et al. (2024).
Data collection	Samples from ridges were collected three times in January (R1), once in April (R2), once in May (R2) and four times in July (R3). Ice cores collected for biogeochemical variables were cut into 10 cm long sections in the field and collected in sterile plastic bags. Biogeochemical variables were, when possible, derived from pooled ice core sections of three replicate cores (R3), and during challenging weather periods (R1 and R2) from single ice cores. The core sections were kept dark and cool, transferred to the lab on board and melted in the dark after the addition of filtered seawater: 50 mL 0.22 µm filtered seawater was added per cm of sea ice thickness, and the sea ice samples melted within 24–36 hours in the dark at around 4°C. When possible, the water (20–30 L) inside the voids, below the ridge and below level ice, was sampled using a manual bilge pump with a silicon tube with a diameter of 20 mm into prewashed polyethylene containers.
Timing and spatial scale	R1 was sampled twice for biological variables on January 10 and 24 at around 87°N in the eastern Amundsen Basin. R2 was sampled twice for physical and biological variables in late April and early May at about 84°N in the Nansen Basin. R3 was sampled four times over a period of about one month (July 2020) when the ice floe drifted from 82 to 80°N, over the Yermak Plateau through the Fram Strait. The relatively easier weather conditions in July allowed higher temporal and spatial sampling resolution of the ridge.
Data exclusions	No data were excluded from the analysis, whenever a subset was used to specifically focus on one season (e.g. summer) it is noted clearly in the manuscript.
Reproducibility	no experimental data
Randomization	no experimental data
Blinding	not applicable

Did the study involve field work? ☒ Yes ☐ No

Field work, collection and transport

Field conditions	The samples were collected during the winter (January), spring (April-May) and summer (July) period. During these three periods 2m air temperatures varied between -35 and +2°C (January: -30 and -22°C; April-May: -18 and -8°C; July: -2 and +2°C; Shupe et al. 2022, Elementa) and surface ocean water temperatures were about -1.7°C (Rabe et al. 2022, Elementa). The three investigated ridges were similar in characteristics. They formed during the MOSAiC drift (similar age) and were composed of thin ice blocks, with similar sail heights (1–2 m) and average keel depths (3.2–4.3 m).
Location	The yearlong MOSAiC ice drift (October 2019 to September 2020), with the research vessel Polarstern serving as the base, started in the eastern Eurasian Basin and crossed the Amundsen and Nansen basins towards the Fram Strait. HAVOC Ridges - Safe HAVens for ice-associated flora and fauna in a seasonally ice-covered Arctic Ocean) specific samples for this study were collected during leg2 (January 2020; 87.176686, 112.755364; 87.4199366, 93.0894339), leg3 (April and May 2020; 84.1200365, 15.9396856; 83.9216011, 18.099531) and leg4 (July 2020; 81.6978255, 6.2498296; 81.4436516, 0.4906923; 80.6054659, -0.5729281; 79.7912909, -1.7608771).

Access & import/export	The MOSAiC Campaign required 10 years of planning and a larger international consortium (20 nations) to allow for a one year drift campaign, thereby allowing to study the central Arctic Ocean in winter time when ice is usually too thick to allow ship-based expedition to reach the area.
Disturbance	*Describe any disturbance caused by the study and how it was minimized.*

Reporting for specific materials, systems and methods

We require information from authors about some types of materials, experimental systems and methods used in many studies. Here, indicate whether each material, system or method listed is relevant to your study. If you are not sure if a list item applies to your research, read the appropriate section before selecting a response.

Materials & experimental systems

n/a	Involved in the study
☒	☐ Antibodies
☒	☐ Eukaryotic cell lines
☒	☐ Palaeontology and archaeology
☒	☐ Animals and other organisms
☒	☐ Clinical data
☒	☐ Dual use research of concern
☒	☐ Plants

Methods

n/a	Involved in the study
☒	☐ ChIP-seq
☐	☒ Flow cytometry
☒	☐ MRI-based neuroimaging

Plants

Seed stocks	n/a
Novel plant genotypes	n/a
Authentication	n/a

Flow Cytometry

Plots

Confirm that:

☒ The axis labels state the marker and fluorochrome used (e.g. CD4-FITC).

☒ The axis scales are clearly visible. Include numbers along axes only for bottom left plot of group (a 'group' is an analysis of identical markers).

☐ All plots are contour plots with outliers or pseudocolor plots.

☒ A numerical value for number of cells or percentage (with statistics) is provided.

Methodology

Sample preparation	Samples for flow cytometric analysis were taken in triplicates or quadruplicates of 1.8 mL of sample water and fixed with 36 µL 25 % glutaraldehyde (0.5 % final concentration) at 4 °C in the dark for approximately 2 hours, then flash frozen in liquid nitrogen and stored at -80 °C until analysis. Prior analysis of bacteria and heterotrophic nanoflagellates (HNF), samples were first thawed, diluted x10 and x100 with 0.2 µm filtered TE buffer (Tris 10 mM, EDTA 1 mM, pH 8), stained with a green fluorescent nucleic acid dye (SYBR Green I ; Molecular Probes, Eugene, Oregon, USA) and then incubated for 10 min at 80°C in a water bath (Marie et al. 1999).
Instrument	FACS Calibur flow cytometer (Becton Dickinson) with a 15 mW 480 nm (blue) laser for bacteria counts.<br>Attune® NxT, Acoustic Focusing Cytometer (Invitrogen by Thermo Fisher Scientific) with a 20 mW 488 nm (blue) laser for counting Autotrophic pico-and nano-sized plankton and HNF.
Software	BD CellQuest Pro (version 6.0) and Attune® NxT software v3.1.2
Cell population abundance	The abundance of bacteria was determined using a FACS Calibur (Becton Dickinson) flow cytometer with a 15 mW 480 nm

Cell population abundance	(blue) laser. Stained samples were counted at a flow rate of around 60 µL min-1 and the different groups discriminated on a biparametric plot of green florescence (BL1) vs. side scatter (SSC). Autotrophic pico-and nano-sized plankton, as well as HNF were counted using the Attune® NxT, Acoustic Focusing Cytometer (Invitrogen by Thermo Fisher Scientific) directly after thawing and the various groups discriminated based on their red fluorescence (BL3) vs. orange fluorescence (BL2), red fluorescence (BL3) vs. side scatter (SSC), HNF: green florescence (BL1) vs. side scatter (SSC) and Synechococcus: orange fluorescence (BL2) vs. side scatter (SSC). No cell sorting was performed.
Gating strategy	Bacteria were discriminated on a biparametric plot of green florescence (BL1) vs. side scatter (SSC). This allowed to distinguish bacteria of different sizes as described in Marie et al. 1999.<br>Names in the published flow cytometry data are in accordance to "Standards and Best Practices For Reporting Flow Cytometry Observations: a technical manual (Version 1.1)" (Neeley et al., 2023).<br>Autotrophic pico-and nano-sized plankton in the size range of 1 to 20 µm were discriminated based on their red fluorescence (BL3) vs. orange fluorescence (BL2), red fluorescence (BL3) vs. side scatter (SSC) and orange fluorescence (BL2) vs. side scatter (SSC). Names of size groups of photosynthetic and heterotrophic organisms are in accordance to "Standards and Best Practices For Reporting Flow Cytometry Observations: a technical manual (Version 1.1)" (Neeley et al., 2023). A short summary is listed here: RedPico = picophytoplankton (1-2 µm); RedNano = Nanophytoplankton (2-20µm), which includes subgroups RedNano_small (2-5 µm), RedNano_large (5-20 µm); OraPico = Nanophytoplankton with more orange fluorescence; OraNano = Cryptophytes; OraPicoProk = Synechococcus. Further, exemplary plots showing the gating strategies that were followed can be found in "Interoperable vocabulary for marine microbial flow cytometry" (Thyssen et al., 2022).<br><br>Neeley, Aimee; Soto, Inia; Proctor, Christopher W (2023): Standards and Best Practices For Reporting Flow Cytometry Observations: a technical manual. Version 1.1. UNESCO/IOC, https://doi.org/10.25607/OBP-1864.2<br>Marie, Dominique; Brussaard, Corina P D; Thyrhaug, Runar; Bratbak, Gunnar; Vaulot, Daniel (1999): Enumeration of Marine Viruses in Culture and Natural. Applied and Environmental Microbiology, 65(1), 45-52, https://doi.org/10.1128/AEM.65.1.45-52.1999<br>Thyssen, Melilotus et al. (2022),: Interoperable vocabulary for marine microbial flow cytometry. Front. Mar. Sci., https://doi.org/10.3389/fmars.2022.975877

☒ Tick this box to confirm that a figure exemplifying the gating strategy is provided in the Supplementary Information.
